# Towards safer childbirth: a journey of a thousand miles

**DOI:** 10.1111/aogs.14381

**Published:** 2022-05-15

**Authors:** Julia Savchenko, Lars Ladfors, Linda Hjertberg, Eric Hildebrand, Sophia Brismar Wendel

**Affiliations:** ^1^ Department of Obstetrics and Gynecology Stockholm South General Hospital (Södersjukhuset) Stockholm Sweden; ^2^ Department of Clinical Science and Education Stockholm South General Hospital (Södersjukhuset) Karolinska Institutet Stockholm Sweden; ^3^ Department of Obstetrics and Gynaecology Institute of Clinical Sciences, Sahlgrenska Academy, Gothenburg University Sahlgrenska University Hospital Gothenburg Sweden; ^4^ Department of Obstetrics and Gynecology Vrinnevi Hospital Norrköping Sweden; ^5^ Department of Obstetrics and Gynecology and Department of Biomedical and Clinical Sciences Linköping University Linköping Sweden; ^6^ Department of Obstetrics and Gynecology Danderyd Hospital Stockholm Sweden; ^7^ Department of Clinical Sciences Karolinska Institutet, Danderyd Hospital Stockholm Sweden

*Sir*,

We were pleased to read the recent Letter to the Editor by José Guida and Maria Costa, who were interested in our publication in AOGS.^1^ In our study, we explored Swedish national data on labor outcomes stratified by the Robson Ten Group Classification System.^2^ This classification was suggested by Michael S. Robson for the analysis of cesarean section rates and is now widely used for this purpose.^3^, ^4^ In our paper we demonstrated how the Robson classification can be applied to a broader set of basic obstetric endpoints for a more balanced and nuanced audit of labor care performance.^2^

In their letter, José Guida and Maria Costa share their insights gained by analysis of their database from a Brazilian maternity hospital where the occurrence of preeclampsia was considered as outcome. Preeclampsia is a potentially life‐threatening condition that has great impact on maternal and infant health. Finding a balance between monitoring of pregnant women for early detection of preeclampsia and timely action to prevent its complications while avoiding overmedicalization and unnecessary interventions can be tricky. We hope that the authors’ observations regarding preeclampsia rates in different groups of women and the high contribution of low‐risk women to the overall preeclampsia rate can be a helpful step in achieving this goal.

However, we find that using preeclampsia as outcome, even if important, can be difficult to interpret. Unlike cesarean section, operative vaginal delivery, obstetric anal sphincter injury, postpartum hemorrhage and low Apgar score, which were suggested as outcomes in our article, the occurrence of preeclampsia is an outcome of pregnancy rather than of labor management. In addition, preeclampsia occurrence is difficult to control. At the same time, neither cesarean section nor operative vaginal delivery is an obvious outcome, even if more traditional. It can be argued that they are both a means and an endpoint, which complicates analysis and goal setting.

We believe that it would be desirable to agree on a minimal set of apparently crucial and easily collectible outcomes of labor management, which will be reported by Robson groups. This will facilitate evidence synthesis and comparisons. Which outcomes should be included in such a set is a matter of further discussion. Of course, researchers should be free to add any outcomes of their interest and perform analyses of any subpopulation they want to study, as for example women with pregestational diabetes, obesity, low socioeconomic status or advanced age.

We thank José Guida and Maria Costa for their interest in our approach and look forward to seeing other articles that apply this method to real‐world data from various regions and populations. We hope this will allow structured comparison and thus promote nuanced discussions on the pros and cons of different obstetric strategies and processes. This could be a next step for further improving labor safety and childbirth experience.
